# Association Between Smoking and Physician-Diagnosed Stroke and Myocardial Infarction in Male Adults in Korea

**DOI:** 10.3390/ijerph13020158

**Published:** 2016-01-25

**Authors:** Sounghoon Chang, Hyeongsu Kim, Vitna Kim, Kunsei Lee, Hyoseon Jeong, Jung-Hyun Lee, Soon-Ae Shin, Eunyoung Shin, Minsu Park, Eunjung Ko

**Affiliations:** 1 Department of Preventive Medicine, School of Medicine, Konkuk University, Seoul 05029, Korea; schang@kku.ac.kr (S.C.); kunsei.lee@kku.ac.kr (K.L.); hspoison@nate.com (H.J.); leedouble@nate.com (J.-H.L.); 2 Department of Dental Hygiene, Suwon Women’s College, Suwon 16632, Korea; kimbit-na@hanmail.net; 3 Big Data Steering Department, National Health Insurance Service, Seoul 04156, Korea; rosa1026@nhis.or.kr; 4 Department of Public Health Administration, Hanyang Women’s University, Seoul 04763, Korea; eyshin@hywoman.ac.kr; 5 Granduate School of Public Health, Inje University, Pusan 50834, Korea; 01023132401@daum.net; 6 Department of Internal Medicine, School of Medicine, Inha University Hospital, Incheon 22212, Korea; eunjjung_86@naver.com

**Keywords:** smoking, stroke, myocardial infarction

## Abstract

To evaluate the association between smoking and physician-diagnosed stroke and myocardial infarction, this study used Community Health Survey data from 2009 on 92,082 males over the age of 30 years. Using multiple logistic regression, association index between smoking and physician-diagnosed stroke and myocardial infarction was calculated after adjusting the effects of age, hypertension, and diabetes. The odds ratios (95% confidence interval) of the physician-diagnosed stroke and myocardial infarction in the smoking group were 1.12 (1.02–1.24) and 1.21 (1.06–1.38) compared to the non-smoking group. The values of the physician-diagnosed stroke and myocardial infarction were 0.84 (0.74–0.94) and 0.96 (0.82–1.12) in the current-smoking subgroup, 1.38 (1.24–1.53) and 1.45 (1.26–1.67) in the ex-smoking subgroup, 1.39 (1.18–1.63) and 1.85 (1.53–2.24) in the 10- to 19-year smokers groups, 1.39 (1.22–1.58) and 1.36 (1.15–1.60) in the 30- to 40-year smokers groups, and 0.53 (0.44–0.63) and 0.47 (0.36–0.63) in those who had smoked for over 50 years. These results showed smoking was a risk factor for stroke and myocardial infarction in Korean males. This objective evidence should guide policy-making and public health interventions in the fields of smoking prevention and prohibition.

## 1. Introduction

Smoking causes and complicates numerous diseases including lung cancer and cardiovascular disease in Surgeon General Report [1], 20-year follow up study [2], 40-year follow up study [3], 50-year follow up study [4], and other studies [5,6,7,8]. Although the smoking rate of Korean males over the age of 19 years old decreased from 66.3% in 1998 to 42.1% in 2013 [9], this rate is still higher than the average smoking rate (25.7%) of males of Organization for Economic Cooperation and Development countries in 2011 and Korea has the highest male smoking rate of all countries [10]. In fact, most smoking-related research in Korea has focused on smoking habits, attitudes toward smoking, factors relevant to smoking, and quitting smoking. Recently, several studies have measured the burden of smoking-related disease and death on Korean society (e.g., medical costs and Disability-adjusted life year) [11,12,13,14,15], but most works have cited results from foreign studies that show the relationships between smoking and disease. Although some studies have explored the correlations between smoking and cancer [16,17], death [18], and cardiovascular disease [19,20] using epidemiological data of Korea, there are still few studies that dealt with relationship between smoking and its effect on disease occurrence such as cardiovascular disease. 

In order to elucidate the relationship between smoking and disease occurrence based on Korean epidemiological data, this study aimed to evaluate the association between smoking and physician-diagnosed stroke and myocardial infarction with the Community Health Survey (CHS).

## 2. Methods

### 2.1. Study Data and Subjects

We analyzed the 2009 CHS data gathered by the Korea Centers for Disease Control and Prevention. The study subjects were 92,082 of the 92,560 males over the age of 30 years; we excluded 478 males who did not respond to questions related to smoking. The 2009 CHS is representative of adults over the age of 19 years old (thus born before 31 July 1990) from each *si*, *gun*, and *gu* (city and town). Resident registration data current as of April 2009 (the latest date for which representative data were available) were used to form the sample frame. 

The CHS had a two-stage sampling process. In the first stage, sample areas (*tong/ban/ri*) were selected. These were the primary sample units, selected on the basis of the number of households in the *dong/eup/myeon* (the smallest administrative units) using a probability proportional to the sampling method. In the second stage, which selected sample households, the number of households in each selected *tong/ban/ri* were identified, to create a house directory, and sample households were selected using systematic sampling methods. These methods were used to ensure that the sample units were representative. To allow comparisons among health centers located all over the country, the error value was set at ±3% and the average sample size was approximately 900 subjects. Professionally-trained interviewers visited all sample households and conducted the CHS through one-on-one personal interviews. Each interview was approximately 45 min long and interviewees answered questions in a self-reporting format contained in a questionnaire. Each interviewee first signed a consent form [21]. If you want to see the full questionnaire and understand the CHS in Korea, you can visit the website [22]. 

### 2.2. Selection and Manipulation of Variables

This study used answers to CHS questionnaire items that were appropriate to the purpose of the study. These included any history and time of diagnosis of physician-diagnosed stroke (PDS) or myocardial infarction (PDMI), data on smoking and age, and any history and time of diagnosis of physician-diagnosed hypertension and/or diabetes. 

#### 2.2.1. Dependent Variables: PDS and PDMI 

A history of stroke or myocardial infarction was identified based on the answers to questions on PDS or PDMI. The time at which a disease was diagnosed by a physician was defined as the time at which the disease occurred.

#### 2.2.2. Independent Variable: Smoking Exposure

Smoking exposure was classified into three subtypes based on the following criteria: (1) whether a subject had smoked to date (non-smoking/smoking); (2) current status of smoking (non-smoking ex-smoking/current smoking); and (3) the period of exposure to smoking (non-smoking/0–9/10–19/20–29/30–39/40–49/over 50 years). Using the CDC’s criteria for smoking [23], non-smoking referred to subjects who had never smoked throughout their lives or those who had smoked less than five packs of cigarettes (100 cigarettes). Smoking included ex-smoking and current smoking. Ex-smoking referred to subjects who had smoked more than five packs of cigarettes (100 cigarettes) throughout their lives but who had quit smoking before the time of the survey, and current smoking referred to subjects who had smoked more than five packs of cigarettes (100 cigarettes) prior to the survey and continued to smoke at the time of the survey. 

The period of smoking of each subject in the current smoking subgroup began at the age at which he started smoking and ran to the time of the survey in cases where disease (stroke or myocardial infarction) had not occurred. In cases where disease had occurred, the period of smoking ran from the age at which the subject started smoking to the time at which disease occurred. The period of smoking of a subject who was classed as ex-smoking began at the age at which he started smoking and ran to the time at which he quit smoking if disease had not occurred. In cases where disease occurred, the period of smoking ran from the age at which a subject started smoking to the time at which he quit smoking, if disease occurred after he quit smoking, but the period of smoking ran from the age at which he started smoking to the time at which disease occurred, if disease occurred before he quit smoking. 

#### 2.2.3. Adjusted Variables: Age, Physician-Diagnosed Hypertension, Physician-Diagnosed Diabetes

The adjusted variables were age (30–39, 40–49, 50–59, 60–69, and above 70 years of age), and physician-diagnosed hypertension and diabetes. Hypertension or diabetes were defined as risk factors for stroke or myocardial infarction in subjects with no history of stroke or myocardial infarction, but with physician-diagnosed hypertension or diabetes, with a history of stroke or myocardial infarction, or who were diagnosed with hypertension or diabetes more than one year prior to a diagnosis of stroke or myocardial infarction. Hypertension or diabetes were considered to not be risk factors for stroke or myocardial infarction in those with no history of stroke, myocardial infarction, hypertension, or diabetes; and in those diagnosed with hypertension or diabetes less than one year before or after a diagnosis of stroke or myocardial infarction.

### 2.3. Data Analysis

Statistical Analysis Software (SAS ver. 9.1, SAS Institute, Cary, NC, USA) was used for data analysis. First, univariate frequency analysis was performed between PDS or PDMI and the smoking exposure variables, age, hypertension, and diabetes. Then multivariate logistic regression analysis was performed using PDS or PDMI as the dependent variable; age, hypertension, and diabetes as adjusted variables; and the smoking exposure variables as independent variables. As a result, we obtained the association index between smoking and physician-diagnosed stroke or myocardial infarction with odds ratios (ORs) and 95% confidence intervals (CIs). Statistical significance was established at a *p*-value of less than 0.05. 

### 2.4. Ethics Statement

This study was reviewed and approved by the Institutional Review Boards of Konkuk University Hospital (approval number: KUH1260020). Because this study used the secondary data of 2009 CHS, we did not have to gather the informed consent from all subjects.

## 3. Results

### 3.1. General Characteristics

In terms of age distribution, males aged 40–49 years were the largest population (25.4%) (Table 1). The proportion of smoking subjects was 77.0% (current smoking 46.8% and ex-smoking 30.2%). In terms of the period of smoking, subjects who had smoked for 10–19 years represented the largest population (22.1%); 3.8% had smoked for more than 50 years. The prevalence of physician-diagnosed disease was 2.6% for stroke, 1.5% for myocardial infarction, 20.7% for hypertension, and 8.7% for diabetes. 

**Table 1:** General characteristics of the study population.

Variable		N (92,082)	% (100.0)
Age (years)	30–39	20,069	21.8
	40–49	23,377	25.4
	50–59	19,776	21.5
	60–69	15,954	17.3
	**≥**70	12,906	14.0
Smoking status	Non-smoking	21,147	23.0
	Smoking	70,935	77.0
	Current smoking	43,088	46.8
	Ex smoking	27,847	30.2
Period of smoking (Years)	Non-smoking	21,147	23.0
	0–9	7531	8.0
	10–19	20,384	22.1
	20–29	19,845	21.6
	30–39	12,645	13.7
	40–49	7150	7.8
	**≥**50	3509	3.8
Physician-diagnosed stroke	Yes	2380	2.6
	No	89,699	97.4
Physician-diagnosed myocardial infarction	Yes	1387	1.5
	No	90,695	98.5
Physician-diagnosed hypertension	Yes	19,040	20.7
	No	72,985	79.3
Physician-diagnosed diabetes	Yes	7991	8.7
	No	84,050	91.3

### 3.2. Prevalence of PDS according to Variables

The prevalence of PDS among smoking subjects was 2.61%, higher than the prevalence of disease among non-smoking subjects (2.50%), but the difference was not significant (*p* = 0.359) (Table 2). Among smoking subjects, the prevalence of PDS was 1.51% in the current smoking subgroup and 4.31% in the ex-smoking subgroup (*p* < 0.001). The values were 1.66% among subjects who had smoked for 0–9 years, 2.24% among those who had smoked for 20–29 years, and 5.24% among those who had smoked for 40–49 years, demonstrating that the prevalence of disease significantly increased as the period of smoking increased (*p* < 0.001). It also increased with age, being 0.10% in subjects in their 30s, 1.82% among those in their 50s, and 8.56% among those aged above 70 years (*p* < 0.001). Finally, the prevalence of PDS among subjects with hypertension as a risk factor was 3.35%, whereas it was 2.39% among subjects without this risk factor (*p* < 0.001). The prevalence of PDS among subjects with diabetes as risk factor was 3.67%. It was 2.48% among subjects without this risk factor (*p* < 0.001).

### 3.3. Association between Smoking and PDS

Table 3 shows the OR values of PDS with reference to each variable of smoking exposure after adjusting for age, hypertension, and diabetes, calculated via multivariate logistic regression analysis. Compared to the non-smoking group, the OR of the PDS in the smoking group was 1.12 (95% CI 1.02–1.24). The values were 0.84 (95% CI 0.74–0.94) in the current smoking subgroup and 1.38 (95% CI 1.24–1.53) in the ex-smoking subgroup. Compared to the group that had never smoked, the values were 1.39 (95% CI 1.18–1.63) in the group that had smoked for 10–19 years, 1.39 (95% CI 1.22–1.58) in those who had smoked for 30–39 years, and 0.53 (95% CI 0.44–0.63) in those who had smoked for more than 50 years.

**Table 2:** Prevalence of physician-diagnosed stroke and myocardial infarction by smoking variables.

		Physician-Diagnosed Stroke			Physician-Diagnosed Myocardial Infarction		
		N	%	*p*-Value	N	%	*p*-Value
Total		2380	2.58		1387	1.51	
Smoking	No	528	2.50	0.359	288	1.36	0.049
	Yes	1852	2.61		1099	1.55	
Smoking status	Non-smoking	528	2.50	<0.001	288	1.36	<0.001
	Current smoking	652	1.51		425	0.99	
	Ex-smoking	1200	4.31		674	2.42	
Period of smoking(Years)	Non-smoking	528	2.50	<0.001	288	1.36	<0.001
	0–9	122	1.66		73	0.99	
	10–19	242	1.19		184	0.90	
	20–29	446	2.24		278	1.40	
	30–39	509	4.01		301	2.38	
	40–49	373	5.24		201	2.81	
	**≥**50	160	4.66		62	1.77	
Physician-diagnosed hypertension	No	1769	2.39	<0.001	1004	1.37	<0.001
	Yes	608	3.35		382	2.05	
Physician-diagnosed diabetes	No	2059	2.48	<0.001	1205	1.43	<0.001
	Yes	283	3.67		180	2.31	
Age (Years)	30–39	21	0.10	<0.001	16	0.08	<0.001
	40–49	146	0.62		91	0.39	
	50–59	359	1.82		286	1.45	
	60–69	749	4.69		514	3.22	
	**≥**70	1105	8.56		480	3.72	

**Table 3:** Association between smoking and physician-diagnosed stroke and myocardial infarction.

		Physician-Diagnosed Stroke			Physician-Diagnosed Myocardial Infarction		
		Odds Ratio	95% ConfidenceInterval of OR		Odds Ratio	95% ConfidenceInterval of OR	
			Lower	Upper		Lower	Upper
Smoking	No	1			1		
	Yes	1.12	1.02	1.24	1.21	1.06	1.38
Smoking status	Non-smoking	1			1		
	Current smoking	0.84	0.74	0.94	0.96	0.82	1.12
	Ex-smoking	1.38	1.24	1.53	1.45	1.26	1.67
Period of smoking(Years)	Non-smoking	1			1		
	0–9	1.02	0.84	1.25	1.11	0.85	1.44
	10–19	1.39	1.18	1.63	1.85	1.53	2.24
	20–29	1.55	1.36	1.78	1.64	1.38	1.94
	30–39	1.39	1.22	1.58	1.36	1.15	1.60
	40–49	0.94	0.82	1.08	0.95	0.79	1.14
	**≥**50	0.53	0.44	0.63	0.47	0.36	0.63

All values were calculated after adjusting the effect of ages, hypertension, and diabetes.

### 3.4. Prevalence of PDMI according to Smoking Variables

The prevalence of PDMI among the smoking group was 1.55%, significantly higher than that of non-smoking subjects (1.36%) (*p* = 0.049) (Table 2). The values were 0.99% in the current smoking subgroup and 2.42% in the ex-smoking subgroup (*p* < 0.001), 2.05% among subjects with hypertension as a risk factor and 1.37% without this risk factor (*p* < 0.001), and 2.31% among subjects with diabetes as a risk factor and 1.43% without this risk factor (*p* < 0.001). Finally, the values were 0.99% in those who had smoked for 0–9 years, 1.40% in smokers for 20–29 years, 2.81% in smokers for 40–49 years; 0.08% among subjects in their 30s, 1.45% in subjects in their 50s, and 3.73% in subjects above 70 years of age. These results indicate that the prevalence of disease significantly increased as the period of smoking increased (*p* < 0.001) and with age (*p* < 0.001).

### 3.5. Association between Smoking and PDMI

Table 3 shows the OR values of the PDMI for each variable of smoking exposure after adjusting for age, hypertension, and diabetes, according to multivariate logistic regression analysis. Compared to the non-smoking group, the OR value of the PDMI in the smoking group was 1.21 (95% CI 1.06–1.38). The values were 0.96 (95% CI 0.82–1.12) in the current smoking subgroup and 1.45 (95% CI 1.26–1.67) in ex-smokers. Compared to the group that has never smoked, the values were 1.85 (95% CI 1.53–2.24), 1.36 (95% CI 1.15–1.60), and 0.47 (95% CI 0.36–0.63) in those who had smoked for 10–19 years, 30–39 years, and >50 years, respectively.

## 4. Discussion

This study used Korean data to evaluate the association between smoking and PDS or PDMI after adjusting for age, hypertension, and diabetes. When smoking exposure was classified in terms of whether a subject had ever smoked, by the current status of smoking (non-smoking, ex-smoking, current smoking), or by the period of smoking, smoking was a risk factor for the development of PDS and PDMI.

Numerous studies involving non-Korean subjects have shown that smoking is a risk factor for stroke and myocardial infarction. In a meta-analysis, the overall relative risk of stroke associated with cigarette smoking was 1.5 (95% CI 1.4–1.6) [24]. In addition, the overall age-adjusted OR of fatal stroke in daily smokers was 3.5 and increased with increasing cigarette consumption [25]. In the Framingham Heart Study cohort, smoking was significantly associated with stroke after age and hypertension were taken into account [26]. The relative risk of stroke in heavy smokers (greater than 40 cigarettes per day) was twice that of light smokers (fewer than 10 cigarettes per day), and the risk in lapsed smokers fell to the same level as that of non-smokers soon after stopping. The risk of stroke increased as the number of cigarettes smoked daily increased. Finally, compared to non-smokers, cigarette smokers had two to three times higher risk for thromboembolic or hemorrhagic stroke, after controlling for age, diastolic blood pressure, coronary heart disease, and other risk factors [27]. In addition, current smoking was associated with a greater risk of non-fatal acute myocardial infarction compared to never-smokers, with the risk increasing by 5.6% for every cigarette smoked [28]. In an epidemiological study conducted in Japan, current smoking was an independent risk factor for acute myocardial infarction after adjusting for hypertension, diabetes, and hypercholesterolemia [29]. Despite the causal relevance of smoking to stroke and myocardial infarction, the precise nature of the toxic components of cigarette smoke, and the mechanisms of cigarette smoking-related cardiovascular dysfunction, remain largely unknown. However, recent experimental and clinical data support the hypothesis that exposure to cigarette smoke increased oxidative stress, in turn potentially initiating cardiovascular dysfunction [30].

A recent cross-sectional study based on data from the Korea National Health and Nutrition Examination Survey showed that, after adjusting for social demographic characteristics, physical condition, and health behavior, the ORs of stroke associated with smoking and myocardial infarction were 1.38 (95% CI 0.99–1.91) and 0.98 (95% CI 0.62–1.56), respectively, showing that smoking was not relevant to the development of these two diseases [19]. The work was meaningful because it addressed the relevance of smoking to cardiovascular disease. However, this study did not take into account the temporal relationship between the start of smoking and disease incidence. Another study that used data on health examinations and insurance claims from the National Health Insurance Service of Korea revealed that smoking was associated with marked increases in the risk for ischemic stroke, subarachnoid hemorrhage, myocardial infarction, and aortic aneurysm, and the effects remained after adjusting for potential confounding factors [20]. 

On the other hand, current smoking was not associated with stroke and myocardial infarction whereas ex-smoking was a significant risk factor for both diseases in our study. This lack of an association with current smoking could be explained by the reverse causation, that is, the development of the diseases is more likely to have induced the smoker to quit the habit. In addition, as the period of smoking increased, smoking generally became a significant risk factor for both diseases, but smoking for over 50 years was not associated with either disease. This means that diseases related to smoking did not occur in those who smoked the most years. Nonetheless, it is known that long-term smokers were at high risk of death caused by diseases related to smoking [4,31,32]. For example, the probability of early death (35–69 years of age) was 24% among non-smokers but 42% among smokers [4]. Ultimately, our results for our longest-smoking subjects reflected selective survival bias (Neyman’s fallacy) [33]. From the point of view of a study design, CHS also has selective survival bias for those who had a fatal stroke or myocardial infarction could not participate in the survey. Those are the first limitation of our study. The other limitations are as follows. Second, as we used secondary data, we could not give adequate consideration to various other factors relevant to the incidence of stroke or myocardial infarction, such as life habits or genetic features of smokers. Third, as the study data were collected using only a questionnaire, the accuracies of the ages at which subjects started to smoke, the period of smoking, and the time at which diseases occurred may not be perfect. In addition, the misclassification of smoking might be differential with regard to the outcome with attendant concerns about the accuracy of the point estimates. Fourth, due to differences in smoking behavior among individuals from different countries, the concentrations of toxic substances in cigarettes, the types of filters used, and the levels of atmospheric and indoor air pollution [34], any direct comparisons of our results to other domestic and international studies on smoking require caution. 

## 5. Conclusions

To evaluate the association between smoking and PDS or PDMI, this study used CHS data from 2009 on 92,082 males over the age of 30 years in Korea. Despite some limitations and the fact that our study was cross-sectional, this study is significant in that it reflects the characteristics of cohort study and elucidates smoking as a risk factor for incidence of stroke and myocardial infarction. Thus, our results provide objective evidence that can be used in policy-making and public health initiatives for smoking prevention and prohibition.

## References

[1] U.S. Department of Health Education and Welfare (1964). Smoking and Health. Report of the Advisory Committee to the Surgeon General of the Public Health Service. http://profiles.nlm.nih.gov/ps/access/nnbbmq.pdf.

[2] Doll R., Peto R. (1976). Mortality in relation to smoking: 20 years’ observations on male British doctors. Br. Med. J..

[3] Doll R., Peto R., Wheatley K., Gray R., Sutherland I. (1994). Mortality in relation to smoking: 40 years' observations on male British doctors. BMJ.

[4] Doll R., Peto R., Boreham J., Sutherland I. (2005). Mortality from cancer in relation to smoking: 50 years observations on British doctors. Br. J. Cancer.

[5] Chang C.M., Corey C.G., Rostron B.L., Apelberg B.J. (2015). Systematic review of cigar smoking and all cause and smoking related mortality. BMC Public Health.

[6] Schane R.E., Ling P.M., Glantz S.A. (2010). Health effects of light and intermittent smoking: A review. Circulation.

[7] Alberg A.J. (2008). Cigarette smoking: Health effects and control strategies. Drugs Today.

[8] U.S. Department of Health and Human Services, Centers for Disease Control and Prevention, National Center for Chronic Disease Prevention and Health Promotion The Health Consequences of Smoking—50 Years of Progress: A Report of the Surgeon General. http://www.surgeongeneral.gov/library/reports/50-years-of-progress/full-report.pdf.

[9] Ministry of Health and Welfare, Korea Centers for Disease Control and Prevention Korea Health Statistics 2013: Korea National Health and Nutrition Examination Survey (KNHANES VI-1). http://www.cdc.go.kr/CDC/intro/CdcKrIntro0201.jsp?menuIds=HOME001-MNU1154-MNU0005-MNU0011.

[10] Organization for Economic Cooperation and Development Health at a Glance 2013: OECD Indicators. http://www.oecd.org/els/health-systems/Health-at-a-Glance-2013.pdf.

[11] Kang H.Y., Kim H.J., Park T.K., Jee S.H., Nam C.M., Park H.W. (2003). Economic burden of smoking in Korea. Tob. Control.

[12] Ha B.M., Yoon S.J., Lee H.Y., Ahn H.S., Kim C.Y., Shin Y.S. (2003). Measuring the burden of premature death due to smoking in Korea from 1990 to 1999. Public Health.

[13] Lee H., Yoon S.J., Ahn H.S. (2006). Measuring the burden of major cancers due to smoking in Korea. Cancer Sci..

[14] Oh I.H., Yoon S.J., Yoon T.Y., Choi J.M., Choe B.K., Kim E.J., Kim Y.A., Seo H.Y., Park Y.H. (2012). Health and economic burden of major cancers due to smoking in Korea. Asian Pac. J. Cancer Prev..

[15] Heo S., Lee J.T. (2015). Disease burdens from environmental tobacco smoke in Korean adults. Int. J. Environ. Health Res..

[16] Park S., Jee S.H., Shin H.R., Park E.H., Shin A., Jung K.W., Hwang S.S., Cha E.S., Yun Y.H., Park S.K. (2014). Attributable fraction of tobacco smoking on cancer using population-based nationwide cancer incidence and mortality data in Korea. BMC Cancer.

[17] Jee S.H., Samet J.M., Ohrr H., Kim J.H., Kim I.S. (2004). Smoking and cancer risk in Korean men and women. Cancer Cause. Control.

[18] Lee E.H., Park S.K., Ko K.P., Cho I.S., Chang S.H., Shin H.R., Kang D., Yoo K.Y. (2010). Cigarette smoking and mortality in the Korean Multi-center Cancer Cohort (KMCC) study. J. Prev. Med. Public Health.

[19] Kim H. (2012). The Correlation between Smoking and Cardiovascular Diseases Based on the 4th and 5th KNHANES.

[20] Lawlor D.A., Song Y.M., Sung J., Ebrahim S., Smith G.D. (2008). The association of smoking and cardiovascular disease in a population with low cholesterol levels: A study of 648,346 men from the Korean national health system prospective cohort study. Stroke.

[21] Kim Y.T., Choi B.Y., Lee K.O., Kim H., Chun J.H., Kim S.Y., Lee D.H., Ghim Y.A., Lim D.S., Kang Y.W. (2012). Overview of Korean community health survey. J. Korean Med. Assoc..

[22] Korea Centers for Disease Control and Prevention Community Health Survey in Korea. http://chs.cdc.go.kr/chs/index.do.

[23] CDC (1994). Cigarette smoking among adults—United States, 1992, and changes in the definition of current cigarette smoking. MMWR.

[24] Shinton R., Beevers G. (1989). Meta-analysis of relation between cigarette smoking and stroke. BMJ.

[25] Håheim L.L., Holme I., Hjermann I., Leren P. (1996). Smoking habits and risk of fatal stroke: 18 years follow up of the Oslo study. J. Epidemiol. Community Health.

[26] Wolf P.A., D'Agostino R.B., Kannel W.B., Bonita R., Belanger A.J. (1988). Cigarette smoking as a risk factor for stroke: Title and subtitle break the framingham study. JAMA.

[27] Nishiyama S., Watanabe T., Arimoto T., Takahashi H., Shishido T., Miyashita T., Miyamoto T., Nitobe J., Shibata Y., Konta T. (2010). Trends in coronary risk factors among patients with acute myocardial infarction over the last decade: the Yamagata AMI registry. J. Atheroscler. Thromb..

[28] Teo K.K., Ounpuu S., Hawken S., Pandey M.R., Valentin V., Hunt D., Diaz R., Rashed W., Freeman R., Jiang L. (2006). Tobacco use and risk of myocardial infarction in 52 countries in the INTERHEART study: A case-control study. Lancet.

[29] Abbott R.D., Yin Y., Reed D.M., Yano K. (1986). Risk of stroke in male cigarette smokers. N. Engl. J. Med..

[30] Thygesen K., Alpert J.S., White H.D. (2007). Universal definition of myocardial infarction. Eur. Heart J..

[31] Zheng W., McLerran D.F., Rolland B.A., Fu Z., Boffetta P., He J., Gupta P.C., Ramadas K., Tsugane S., Irie F. (2014). Burden of total and cause-specific mortality related to tobacco smoking among adults aged ≥45 years in Asia: A pooled analysis of 21 cohorts. PLoS Med..

[32] Yoon S.J., Ha B.M., Kang J.W., Jang H.C. (2001). Estimation of attributable burden due to premature death from smoking in Korea. Korean J. Prev. Med..

[33] Delgado-Rodrı’guez M., Llorca J. (2004). Bias. J. Epidemiol. Community Health.

[34] Warner K.E. (2005). The role of research in international tobacco control. Am. J. Public Health.

